# Stabilization of HIF-2α impacts pancreas growth

**DOI:** 10.1038/s41598-018-32054-5

**Published:** 2018-09-12

**Authors:** Alvaro Flores-Martínez, Alejandro García-Núñez, Anabel Rojas, David A. Cano

**Affiliations:** 1 Unidad de Gestión de Endocrinología y Nutrición. Instituto de Biomedicina de Sevilla (IBiS), Hospital Universitario Virgen del Rocío/CSIC/Universidad de Sevilla, Sevilla, Spain; 2 Centro Andaluz de Biología Molecular y Medicina Regenerativa CABIMER- Universidad Pablo de Olavide- Universidad de Sevilla- Consejo Superior de Investigaciones Científicas (CSIC), Sevilla, Spain; 3 Centro de Investigación Biomédica en Red de Diabetes y Enfermedades Metabólicas Asociadas (CIBERDEM), Madrid, Spain

## Abstract

Hypoxia inducible factors (HIFs) are critical regulators of the response to oxygen deficiency by activating target genes involved in a variety of biological functions. HIFs have been implicated in the pathophysiology of numerous pathologies including cancer. Patients with mutations in the von Hippel-Lindau (VHL) gene, an essential regulator of HIF activity, develop tumors in several organs including the pancreas. Previous functional studies of HIF activation in the pancreas have used *Vhlh* (the murine homolog of *VHL*) deficient mice. However, the role of each specific HIF transcription factors in the pancreas has not been thoroughly examined. We derived mice that constitutively express a normoxia-stable form of HIF2α in the pancreas. Activation of HIF2α in the pancreas severely impairs postnatal exocrine pancreas. Mice with pancreas-specific activation of HIF2α develop histological features reminiscent of pancreatitis including loss of acinar cells, ductal dilation and fibrosis. Moreover, we provide evidence that signaling pathways important for acinar cell homeostasis are altered in HIF2α-overexpressing pancreata.

## Introduction

Hypoxia inducible factors (HIFs) are critical regulators of the response to oxygen deficiency by activating target genes involved in a variety of biological functions including energy metabolism, proliferation, apoptosis, and angiogenesis^1,2^. HIFs are heterodimeric transcription factors, comprising a constitutively expressed HIFβ subunit and an oxygen-regulated HIFα subunit. Three isoforms of HIFα have been identified (HIF1α, HIF2α, and HIF3α) being HIF1α and HIF2α the most extensively studied. Under normal oxygen conditions, prolyl hydroxylases hydroxylate specific proline residues of HIFα subunits^3,4^. HIFα subunits are thus recognized by the protein von Hippel–Lindau (pVHL) and consequently targeted for degradation by the ubiquitin-proteosome pathway^5,6^. Under hypoxia, HIFα is not degraded and thus translocates to the nucleus activating the expression of downstream target genes.

HIFs and the HIF signaling pathway have been implicated in the pathophysiology of numerous pathologies, most prominently cancer^1,7^. The involvement of the HIF pathway in disease is well illustrated by the von Hippel-Lindau (VHL) disease, caused by a germline mutation in the *VHL* gene^8,9^. In the absence of pVHL, HIFα-target genes are aberrantly activated despite normal oxygen levels. Patients with VHL disease develop benign and malignant tumors in multiple organs including the central nervous system, kidney and pancreas^8,9^. Thus, about 70% of VHL patients develop pancreatic pathologies including neuroendocrine tumors, simple cysts and serous microcystic adenomas^10,11^.

The role of HIFs in normal and pathological conditions of the pancreas has recently gained considerable interest. Several studies have revealed that both impairment and activation of the HIF pathway results in pancreatic endocrine dysfunction highlighting the crucial role of this pathway for proper endocrine function^12–18^. Also, HIFs activation has been reported in pancreatitis and pancreatic cancer^19,20^. The effects of HIF activation on pancreas formation have been mostly evaluated through pancreas-specific inactivation of *Vhlh* (the murine homolog of *VHL*) in mice. *Vhlh* mutant mice are born without any apparent pancreatic abnormalities. However, *Vhlh* mutant mice exhibit elevated perinatal lethality due to defects in pancreatic endocrine function^15,21^. The very few surviving *Vhlh* mutant mice develop pancreatic lesions reminiscent of the pancreatic manifestations in VHL patients, although only after a long latency (more than 16 months)^21^.

Other pVHL functions independent of HIFs have been described. These include, among others, regulation of apoptosis, cell adhesion and maintenance of primary cilium^22^. Here, we aim to directly examine the effect of the activation of HIF pathway on pancreas formation. To this end, we derived mice that constitutively express HIF2α in the pancreas. We focused on HIF2α given the prominent role of this HIF factor on proliferation, apoptosis and tumor formation^23^. Also, HIF2α appears to play a critical role in pancreas embryonic development^24^. We show that activation of HIF2α in the pancreas severely impairs postnatal exocrine homeostasis. Mice with pancreas-specific activation of HIF2α display histological features reminiscent of pancreatitis including loss of acinar cells, ductal dilation and fibrosis. Moreover, we provide evidence that signaling pathways important for acinar cell homeostasis are altered in HIF2α-overexpressing pancreas.

## Results

### Specific activation of HIF2α in the pancreas

**Figure 1:**
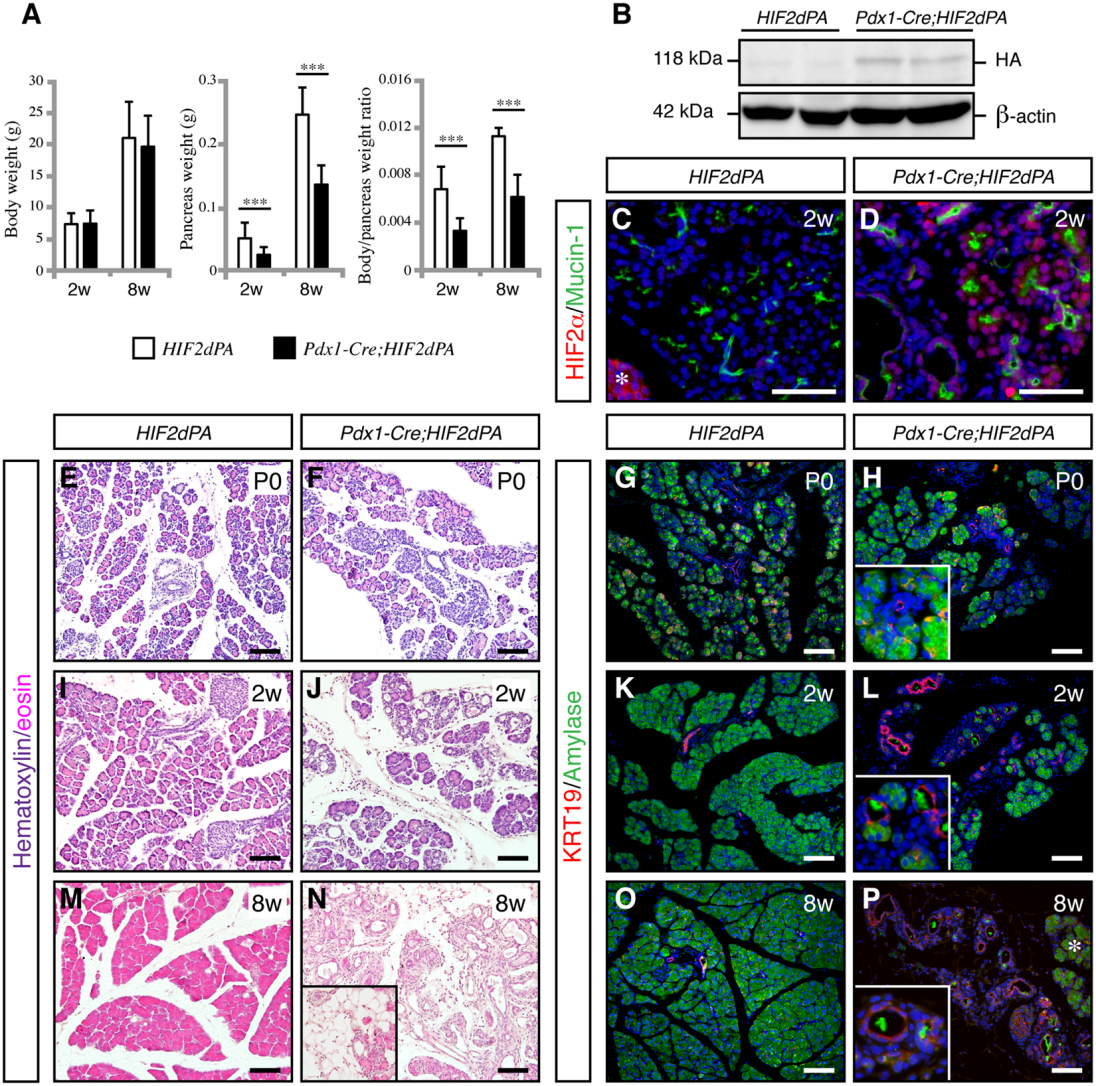
HIF2α stabilization results in exocrine cell atrophy and expansion of duct-like tubular structures. (**A**) Body weight (left panel), pancreas weight (middle panel) and body/pancreas weight ratio (right panel) in *Pdx1-Cre;HIF2dPA* and control mice at 2 and 8 weeks of age. Data are presented as mean ± SD. (**B**) HIF2α accumulation in *Pdx1-Cre;HIF2dPA* analyzed by Western blot with anti-HA antibody. Two independent two-week-old control and mutant mice are shown. ß-actin protein was used for loading control. Full-length blots are presented in Supplementary Fig. 2. (**D**) Immunofluorescence analysis of HIF2α in two-week-old control pancreata. Endogenous HIF2α expression is observed in islets (marked by an white asterisk) but not in exocrine tissue. (**D**) Robust HIF2α accumulation in the pancreas of two-week-old *Pdx1-Cre;HIF2dPA* mice. Hematoxylin/Eosin-stained pancreatic sections from P0 (**E**,**F**), two- (**I**,**J**) and eight-week-old (**M,N**) *Pdx1-Cre;HIF2dPA* and control mice. Inset in **N** shows an area with adipose tissue in *Pdx1-Cre;HIF2dPA* pancreata. Immunofluorescence of amylase and KRT19 shows no differences between *Pdx1-Cre;HIF2dPA* and control mice at P0 (**G**,**H**). Duct-like tubular structures and loss of amylase immunoreactivity in two- (**K**,**L**) and eight-week-old (**O**,**P**) *Pdx1-Cre;HIF2dPA* mice compared to control mice. Note areas with normal acini in 8-week-old *Pdx1-Cre;HIF2dPA* mice (white asterisk in **O**). Insets in (**H**,**L** and **P**) show higher magnification pictures. DAPI staining is shown in blue in (**C**,**D**,**G**,**H**,**K**,**L**,**O** and **P**). Scale bars = 50 µm for (**C**,**D**); 100 µm for (**E**–**P**). ***P < 0.001.

To investigate the role of HIF2α in pancreas formation, we specifically activated HIF2α in the pancreas by crossing mice expressing Cre recombinase under control of the pancreatic and duodenal homeobox 1 (*Pdx1*) promoter (*Pdx1-Cre* mice)^25^ with *HIF2dPA* mice (*Pdx1-Cre;HIF2dPA* mice). In *HIF2dPA* mice^26^, a form of human HIF2α that escapes recognition by the von Hippel-Lindau tumor suppressor protein (i.e. it is not degraded under normal oxygen conditions) is expressed upon Cre recombination. *Pdx1-Cre;HIF2dPA* mice were born at the expected Mendelian ratio and reached adulthood without any sign of compromised health. No differences in total body weight were found between *Pdx1-Cre;HIF2dPA* mice and littermate control mice at both 2 and 8 weeks of age (Fig. 1A). However, a substantial reduction in total pancreatic mass of *Pdx1-Cre;HIF2dPA* mice was found as early as 2 weeks of age, even when pancreatic weight was normalized to body weight (Fig. 1A). Western Blot (Fig. 1B) and immunofluorescence analysis (Fig. 1D) confirmed the efficient accumulation of HIF2α in *Pdx1-Cre;HIF2dPA* pancreas. In control pancreas, endogenous HIF2α expression was found in islets but not in the exocrine compartment (Fig. 1C), as previously reported^20^.

### Exocrine cell atrophy and ductal dilation in postnatal *Pdx1-Cre;HIF2dPA* pancreata

Gross morphology inspection and analysis of Haematoxylin and Eosin-stained tissue of newborn (P0) *Pdx1-Cre;HIF2dPA* pancreata did not reveal any apparent defects (Fig. 1E,F). However, loss of acinar cells and increased dilation of duct-like structures was observed at 2 weeks of age (Fig. 1I,J). These lesions rapidly progressed, and by 8 weeks of age most of the *Pdx1-Cre;HIF2dPA* acinar tissue had been replaced by duct-like tubular structures, stroma and adipose tissue (Fig. 1M,N). To further characterize the pancreatic exocrine defects of *Pdx1-Cre;HIF2dPA* mice we performed double immunohistochemistry for a ductal marker, duct-specific cytokeratin 19 (KRT19), and an acinar cell marker, amylase. The *Pdx1-Cre;HIF2dPA* pancreata appeared normal at P0 (Fig. 1G,H) but beginning at 2 weeks of age amylase expression was substantially reduced concomitant with expansion of duct-like tubular structures (Fig. 1K,L). The acinar architecture appeared disorganized in two-week-old *Pdx1-Cre;HIF2dPA* mice, marked by the loss of the typical round morphology of the acini as well as the dilation of the intracinar lumen (Fig. 1L) and concomitant increase of KRT19-positive epithelial cells. This acinar-ductal metaplasia, broadly defined as replacement of acinar cells by ductal-like cells, did not seem to involve transdifferentiation of acinar cells into ductal-like cells since intermediate cells co-expressing acinar and ductal markers were not observed (Fig. 1L and Supplementary Fig. 1). By 8 weeks of age, the *Pdx1-Cre;HIF2dPA* pancreata appeared severely atrophic with extensive loss of acinar cells and dilated ducts; only residual clusters of acinar cells could be observed (Fig. 1O,P). Islets of *Pdx1-Cre;HIF2dPA* mice displayed a morphology similar to those observed in control mice (Supplementary Fig. 1). Indeed, blood glucose levels in *Pdx1-Cre;HIF2dPA* mice were normal compared to control mice (Supplementary Fig. 1). These results indicate that ectopic HIF2α stabilization in the pancreas leads to extensive loss of acinar cells and expansion of duct-like cells.

### HIF2α activation leads to pancreatic abnormalities reminiscent of pancreatitis

**Figure 2:**
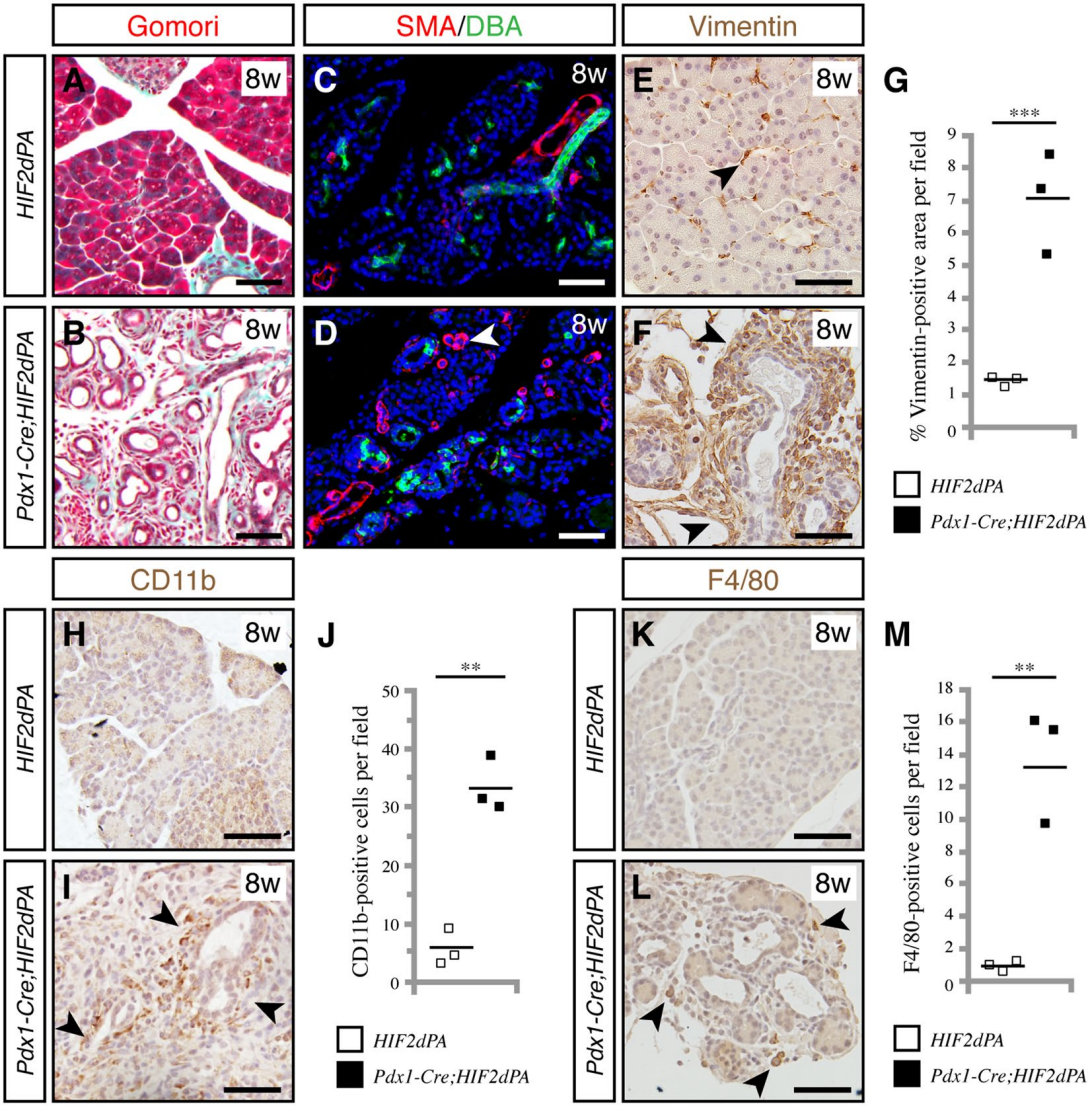
HIF2α stabilization causes pancreatic abnormalities reminiscent of pancreatitis. Gomori trichome staining reveals increased connective tissue (stained in blue) in *Pdx1-Cre;HIF2dPA* pancreata (**B**) compared to control pancreata (**A**) at 8 weeks of age. Increase in smooth muscle actin (SMA) positive cells (white arrowhead) in *Pdx1-Cre;HIF2dPA* pancreata (**D**) compared to control pancreata (**C**) at 8 weeks of age. Ducts are marked by staining with lectin *Dolichos biflorus* agglutinin (DBA). Increase of the mesenchymal marker vimentin in *Pdx1-Cre;HIF2dPA* pancreata (**F**, black arrowheads) at 8 weeks of age. Only a few scattered cells are positive for vimentin in control pancreata (**E**, black arrowhead). (**G**) Quantification of vimentin-positive area per total pancreatic area. CD11b immunohistochemistry in control (**H**) and *Pdx1-Cre;HIF2dPA* pancreata (**I**, black arrowheads) at eight weeks of age. (**J**) Quantification of CD11b-positive cells per field. F4/80 immunohistochemistry in control (**K**) and *Pdx1-Cre;HIF2dPA* pancreata (**L**, black arrowheads) at eight weeks of age. (**M**) Quantification of F4/80-positive cells per field. Data points represent values for each individual mouse. The mean value is indicated as a horizontal line. Scale bars = 50 µm. **P < 0.01; ***P < 0.001.

Loss of acinar cells and ductal expansion are histological hallmarks of pancreatitis. We examined whether other classical features of pancreatitis were also present in *Pdx1-Cre;HIF2dPA* postnatal pancreata. Haematoxylin and Eosin staining showed increased stroma in 8-week-old *Pdx1-Cre;HIF2dPA* pancreata (Fig. 1M,N). Gomori trichome staining revealed an marked increase in connective tissue in *Pdx1-Cre;HIF2dPA* mice (Fig. 2A,B). *Pdx1-Cre;HIF2dPA* pancreatic tissue exhibited a substantial increase in cells expressing smooth muscle actin (SMA), a fibroblast activation marker (Fig. 2C,D) and vimentin, a mesenchymal marker (Fig. 2E–G), thus confirming the formation of pancreatic fibrosis. An increase in inflammatory cells was also apparent in 8-week-old *Pdx1-Cre;HIF2dPA* mice, as evidenced by CD11b immunohistochemistry, a marker of leukocytes (macrophages, neutrophils and granulocytes) (Fig. 2H–J) and F4/80 immunohistochemistry, a specific marker of macrophages (Fig. 2K–M). Thus, HIF2α activation results in fibrosis and increased inflammatory cells in the pancreas, histological features of pancreatitis.

### Increased acinar cell apoptosis and proliferation in *Pdx1-Cre*;*HIF2dPA* mice

**Figure 3:**
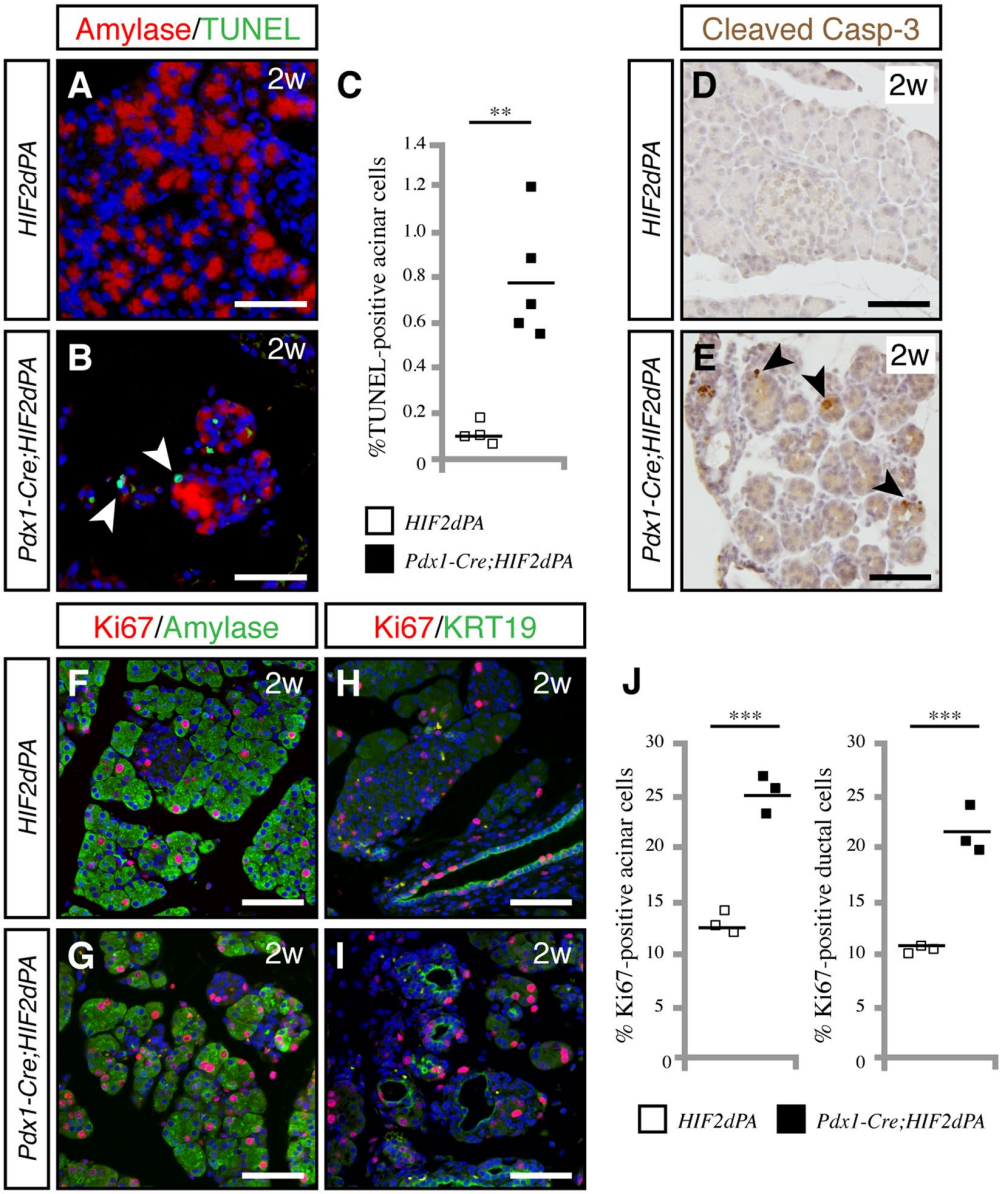
Increased acinar cell apoptosis and proliferation in HIF2α-overexpressing pancreata. (**A**,**B**) TUNEL staining shows an increase in apoptotic acinar cells in the pancreas of two-week-old *Pdx1-Cre;HIF2dPA* mice (**B**, white arrowheads) compared with wild-type (**A**) mice. (**C**) Quantification of apoptotic cells measured as TUNEL positive-cells per total number of amylase-positive cells. Individual data points are presented. The mean value is indicated as a horizontal line. (**D**,**E**) Immunohistochemistry for cleaved caspase-3 confirms the increase in apoptotic cells in *Pdx1-Cre;HIF2dPA* pancreata (**E**, black arrowheads) compared to control pancreata (**D**). Increased acinar cell proliferation in two-week-old *Pdx1-Cre;HIF2dPA* pancreata (**G**) compared to control pancreata (**F**), as shown by double amylase/Ki-67 immunofluorescence. Increased ductal cell proliferation in two-week-old *Pdx1-Cre;HIF2dPA* pancreata (**I**) compared to control pancreata (**H**), as shown by double KRT19/Ki-67 immunofluorescence. (**J**) Quantification of acinar (left graph) and ductal (right graph) proliferating cells, measured as the average number of Ki67-positive cells per amylase- or KRT19-positive cells. Data points represent values for each individual mouse. The mean value is indicated as a horizontal line. Scale bars = 50 µm for **A**,**B**,**D** and **E**; 100 µm for **F–I**. **P < 0.01; ***P < 0.001.

To elucidate the underlying cause of the massive acinar loss in *Pdx1-Cre;HIF2dPA* mice, we assessed acinar cell proliferation, apoptosis and differentiation in two-week-old mice, an age in which *Pdx1-Cre;HIF2dPA* pancreata exhibit areas with preserved acinar tissue. Apoptosis was measured using TUNEL assay. *Pdx1-Cre;HIF2dPA* pancreata showed a marked increase in apoptotic cells compared to control pancreata (Fig. 3A–C). Immunohistochemistry for cleaved caspase-3 confirmed the increase in apoptotic cells of *Pdx1-Cre;HIF2dPA* pancreata (Fig. 3D,E). Cell proliferation was assessed by Ki-67 immunohistochemistry. Control mice displayed substantial pancreatic cell proliferation, as expected at this early postnatal age. However, both acinar (Fig. 3F,G,J) and ductal cell (Fig. 3H,I,J) proliferation was significantly higher in *Pdx1-Cre;HIF2dPA* pancreata. Thus, despite extensive acinar cell loss, pancreatic cell proliferation was prominent in *Pdx1-Cre;HIF2dPA* mice.

### Remodeling of pancreatic acini upon HIF2α activation

**Figure 4:**
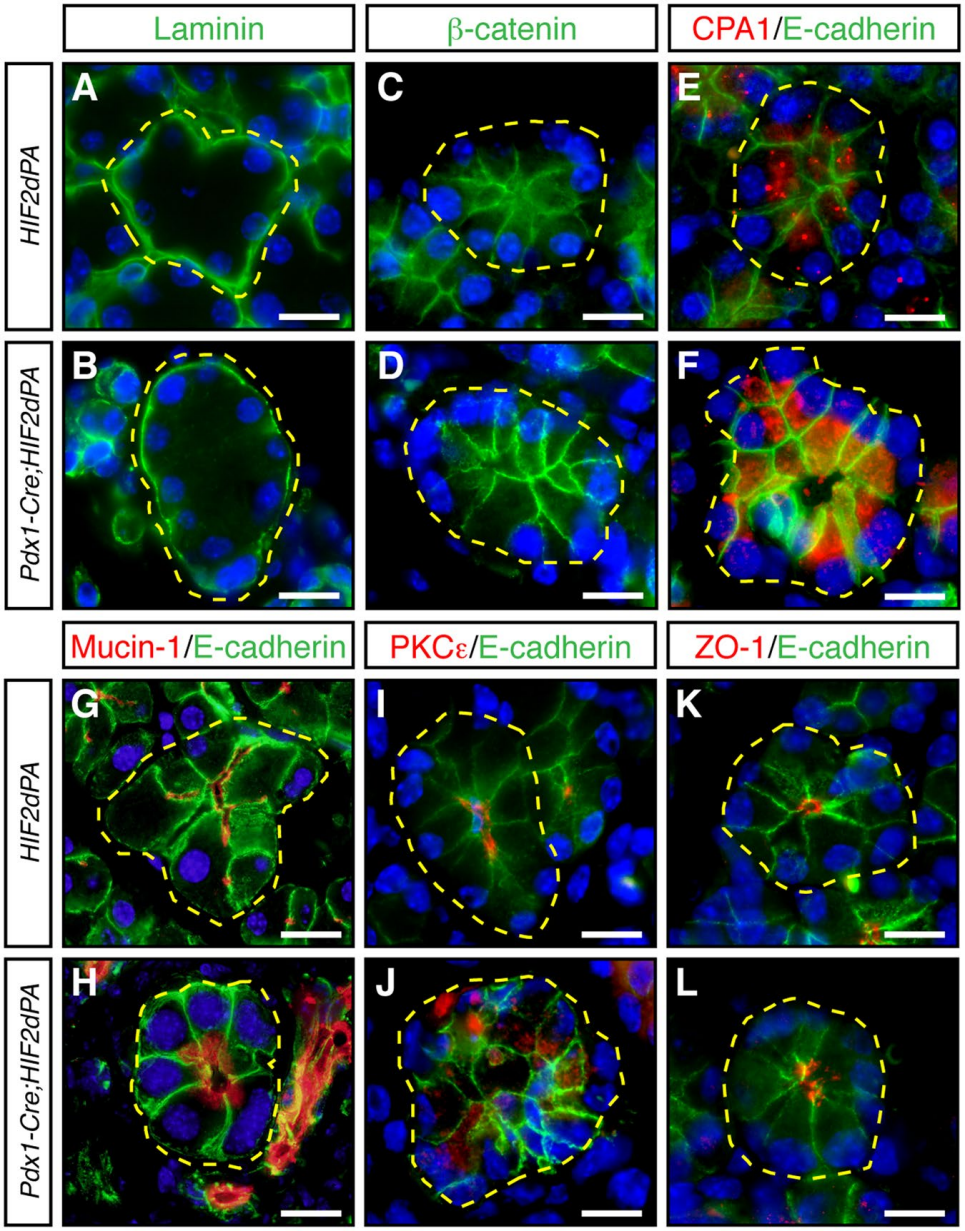
Acini disorganization in HIF2α-overexpressing mice. No changes in the expression or localization of laminin (**A**,**B**), β-catenin (**C**,**D**), and E-cadherin (**E**–**L**) between acini of two-week-old *Pdx1-Cre;HIF2dPA* and control mice. Carboxypeptidase A1 (CPA1) is localized close to the lumen of control acini (**E**). CPA1 does not show a clear apical localization in *Pdx1-Cre;HIF2dPA* acinar cells (**F**). Intracellular accumulation of mucin-1 (**H**), and PKCε (**J**), in *Pdx1-Cre;HIF2dPA* acini while markers are normally localized at the apical region in control acini (**G**,**I**). Zonula occludens 1 (ZO-1) immunofluorescence show lumen dilation in *Pdx1-Cre;HIF2dPA* (**L**) acini compared to control acini (**K**). Nuclei are stained with DAPI (blue). Individual acini are outlined in yellow. Scale bars = 20 µm.

Although the *Pdx1-Cre;HIF2dPA* pancreata appeared largely unaffected at birth, a substantial replacement of acinar cells by cells expressing ductal markers was observed as early as two weeks of age. We analyzed whether acinar cell dedifferentiation could also play a role in this postnatal exocrine pancreatic degeneration. Close examination of acini in two-week-old *Pdx1-Cre;HIF2dPA* mice revealed that dilation of intracinar ducts was associated with reduction of amylase immunoreactivity (Fig. 1L and Supplementary Fig. 1). A key characteristic of the mature exocrine pancreas is the architecture of acini consisting of individual acinar cells that exhibit apical-basal cellular polarity and secrete digestive enzymes into a central lumen. Disruption of acinar cell organization and apical-basal polarity has been associated with postnatal pancreatic exocrine degeneration in various mutant mouse strains^27–29^. Thus, we decided to analyze the accumulation of several markers of acinar cell organization in *Pdx1-Cre;HIF2dPA* mice at two weeks of age when substantial acinar tissue was still preserved. The localization of the basal marker laminin (Fig. 4A,B) as well as basolateral markers β-catenin (Fig. 4C,D) and E-cadherin (Fig. 4E–L) was not largely affected in *Pdx1-Cre;HIF2dPA* acini. In control acinar cells, carboxypeptidase A1 (CPA1) is localized close to the lumen of the acini (Fig. 4E). However, CPA1 did not show a clear apical localization in *Pdx1-Cre;HIF2dPA* acinar cells (Fig. 4F). Immunofluorescence for apical markers mucin-1 (Fig. G,H) and PKCε (Fig. 4I,J) confirmed the mislocalization of apical markers in *Pdx1-Cre;HIF2dPA* mice. Mucin-1 and PKCε were localized exclusively at the apical surface of control acinar cells (Fig. 4G,I). While these proteins still displayed an apical localization in *Pdx1-Cre;HIF2dPA* acinar cells, a marked cytoplasmic accumulation was observed (Fig. 4H,J). Immunofluorescence for mucin-1 also revealed the dilation of acini lumens in *Pdx1-Cre;HIF2dPA* mice (Fig. 4H) compared to the small lumens found in control acini (Fig. 4G). The increase in lumen size of *Pdx1-Cre;HIF2dPA* acini was confirmed by immunofluorescence analysis of the tight junction protein ZO-1 (Fig. 4K,L). Therefore, HIF2α stabilization leads to extensive acini disorganization including intracinar lumen dilation and mislocalization of apical markers while basal markers are appropriately localized.

### Ductal metaplasia and increased vascularization in *Pdx1-Cre;HIF2dPA* pancreata

**Figure 5:**
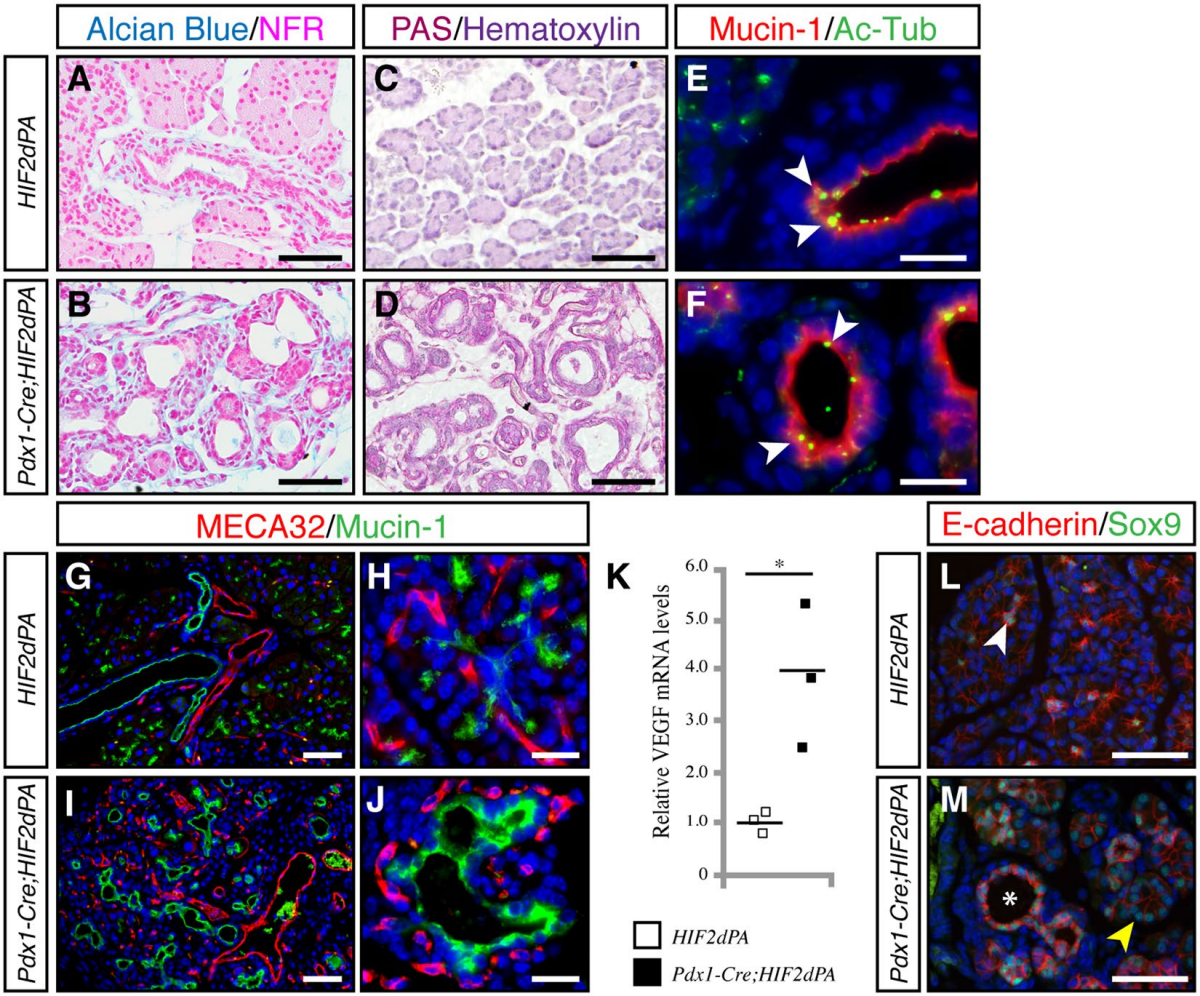
Increased ductal-like structures and vascularization in HIF2α-overexpressing mice. Staining for Alcian Blue/nuclear fast red (NFR) (**A**,**B**) and PAS (**C**,**D**) in *Pdx1-Cre;HIF2dPA* (**B**,**D**) and control pancreata (**A**,**C**) at 2 weeks of age. (**E**,**F**) Primary cilia, marked by acetylated tubulin (Ac-Tub, white arrowheads) are present in pancreatic ducts of control mice (**E**) and duct-like tubular structures of *Pdx1-Cre;HIF2dPA* mice (**F**). Increased vascularization (marked by MECA32 immunostaining) in two-week-old *Pdx1-Cre;HIF2dPA* pancreata (**I**,**J**) compared to control pancreata (**G**,**H**). Note MECA32-positive endothelial cells surrounding duct-like structures in *Pdx1-Cre;HIF2dPA* pancreas (**J**). (**K**) qPCR analysis of VEGF expression in two-week-old pancreata. Data points represent values for each individual mouse. The mean value is indicated as a horizontal line. In two-week-old control pancreata, Sox9 is expressed in scattered centroacinar cells (**L**, white arrowhead). Strong Sox9 expression is found in disorganized acini (**M**, yellow arrowhead) as well as in duct-like tubular structures (**M**, asterisk). Nuclei are stained with DAPI (blue) in (**E**–**J** and **L**,**M**). Scale bars = 50 µm for (**A**–**D**,**G**,**I**,**L** and **M**); 20 µm for (**E**,**F**,**H** and **J**). *P < 0.05.

One of the most notable features of *Pdx1-Cre;HIF2dPA* pancreata was the marked increase in duct-like tubular structures. VHL patients commonly exhibit pancreatic lesions that include cysts and microcystic adenomas^10,11^. These lesions display prominent fibrous stroma and endothelial cells^11^. Also, epithelial cells lack the characteristic papillary structures and mucin found in pancreatic mucinous cystic neoplasms^11^. Similar to the pancreatic lesions of VHL patients, the duct-like tubular structures of *Pdx1-Cre;HIF2dPA* pancreata did not exhibit mucin-producing papillary structures, as revealed by alcian blue (Fig. 5A,B) and PAS staining (Fig. 5C,D). Indeed, *Pdx1-Cre;HIF2dPA* duct-like epithelial cells expressed markers of ductal cells including KRT19 (Fig. 1L,P) and duct binding lectin *Dolichos biflorus* agglutinin (Fig. 2C,D). Also, cilia, which are normally present in control ductal cells^30,31^ but not in pancreatic intraepithelial neoplasia (PanIN) lesions and pancreatic cancer cells^32^, were found in pancreatic duct-like epithelial cells of *Pdx1-Cre;HIF2dPA* mice (Fig. 5E,F). *Pdx1-Cre;HIF2dPA* pancreata displayed increased vascularization (Fig. 5G–K). MECA32-positive endothelial cells were closely intermixed with mucin-1-positive epithelial cells (Fig. 5I,J), as described in pancreatic cysts of VHL patients^11^. In agreement with the increased vascularization, an upregulation of the expression of the HIF-target gene VEGF was found in *Pdx1-Cre;HIF2dPA* pancreata (Fig. 5K). Thus, HIF2α stabilization in the pancreas leads to the formation of pancreatic lesions similar to those found in the pancreas of VHL patients.

### Altered signaling pathways in exocrine pancreas upon HIF2α activation

**Figure 6:**
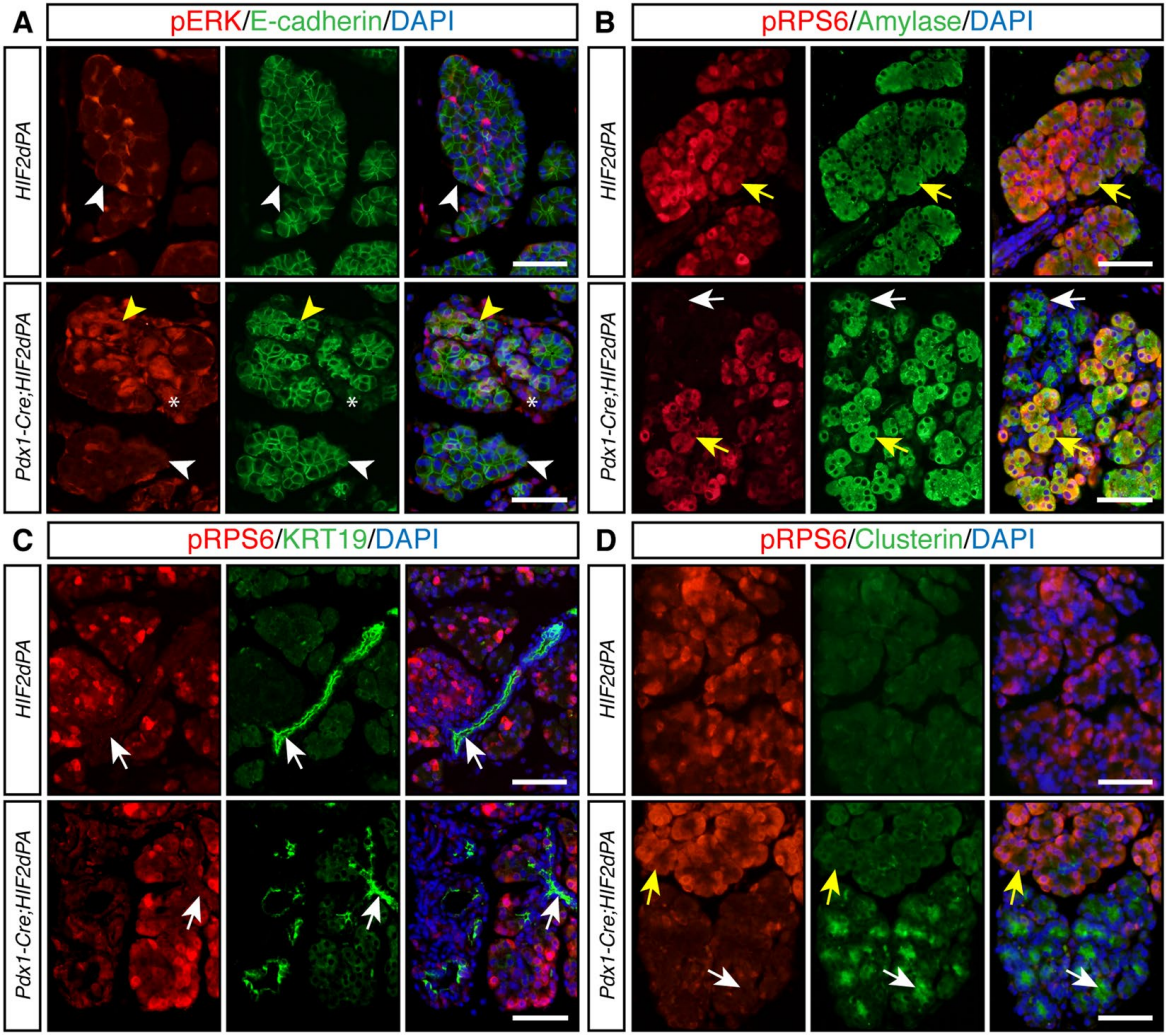
Altered components of MAPK and AKT/mTOR signaling pahtways in HIF2α-overexpressing pancreata. (**A**) Periacinar cells positive for phosphorylated-ERK (pERK) are found in 2-week-old control pancreata but acinar cells are negative for pERK (white arrowheads, top panels). Increased pERK accumulation is observed in 2-week-old *Pdx1-Cre;HIF2dPA* pancreata (yellow arrowheads, bottom panels). Note that the increased expression was limited to areas with acinar-ductal metaplasia (yellow arrowheads, bottom panels). Acini with preserved morphology do not show extensive pERK accumulation (white arrowheads, bottom panels). pERK-positive stromal cells are also observed in *Pdx1-Cre;HIF2dPA* pancreata (asterisks, bottom panels). E-cadherin immunofluorescence was performed to visualize the pancreatic epithelium. (**B**) Double immunofluorescence analysis of phosphorylated RSP6 (pRSP6) and amylase in 2-week-old mice. pRPS6 (yellow arrows) is detected in amylase-positive acinar cells of control pancreata (top panels). In *Pdx1-Cre;HIF2dPA* pancreata, pRPS6 is detected in well-preserved acini (bottom panels, yellows arrows). However, areas with acinar-ductal metaplasia display decreased expression of pRPS6 (white arrows). (**C**) Double immunofluorescence analysis of pRSP6 and KRT19 in 2-week-old mice. Ductal cells (white arrows) are not positive for pRPS6 in both control pancreata (top panels) and *Pdx1-Cre;HIF2dPA* pancreata (bottom panels). (**D**) Clusterin is not expressed in control pancreas (top panels). Areas with acinar-ductal metaplasia in *Pdx1-Cre;HIF2dPA* pancreata display decreased expression of pRPS6 and activation of clusterin expression (white arrows). Yellow arrows indicate acini with normal morphology. Nuclei are stained with DAPI (blue). Scale bars = 50 µm.

The expression of ductal transcription factors such as Sox9 is increased during acinar-ductal metaplasia in models of pancreatic injury^33–35^. In agreement with this, we observed prominent Sox9 expression in both disorganized acini and well-defined duct-like tubular structures of *Pdx1-Cre;HIF2dPA* pancreata (Fig. 5L,M). The MAPK signaling pathway plays an important role in acinar cell homeostasis in response to injury. During pancreatitis the MAPK pathway is up-regulated^36,37^ and it has been shown to be necessary for acinar-ductal metaplasia^38,39^. We evaluated activation of the MAPK pathway, as assessed by immunofluorescence of phosphorylated ERK (pERK). In control pancreata, only a few scattered cells were positive for pERK (Fig. 6A, top panels). In stark contrast, strong nuclear pERK signal was observed in *Pdx1-Cre;HIF2dPA* acinar cells. This activation of MAPK pathway was largely limited to disorganized acini (Fig. 6A, bottom panels), the preserved acini in *Pdx1-Cre;HIF2dPA* mice did not shown substantial pERK signal (Fig. 6A, bottom panels). Of note, prominent MAPK activity was also observed in the stromal cells of *Pdx1-Cre;HIF2dPA* pancreata (Fig. 6A, bottom panels). The AKT/mTOR pathway has also been implicated in acinar-ductal metaplasia^40–42^. Thus, we evaluated the phosphorylation status of the ribosomal protein S6 (RPS6), a downstream target of the AKT/mTOR signaling pathway. As previously shown^42,43^, RPS6 was phosphorylated in acinar (Fig. 6B, top panels), but not ductal cells of control pancreata (Fig. 6C, top panels). In the pancreas of *Pdx1-Cre;HIF2dPA* mice, phosphorylated RPS6 (pRPS6) was easily detected in well-preserved acini (Fig. 6B, bottom panels). However, a dramatic decrease in pRPS6 was observed in areas with disorganized acini (Fig. 6B, bottom panels). Interestingly, we observed an induction of the stress protein clusterin in disorganized acini (Fig. 6D, bottom panels). No expression of clusterin was detected in normal acini of *Pdx1-Cre;HIF2dPA* mice (Fig. 6D, bottom panels) as well as in acini of control mice (Fig. 6D, top panels). Thus, HIF2α activation in the pancreas affects signaling pathways important for exocrine pancreas homeostasis.

## Discussion

Here, we show that specific activation of HIF2α in the pancreas severely disrupts homeostasis of the exocrine pancreas. We derived transgenic mice that specifically expressed a normoxia-stable form of HIF2α in the pancreas. Continuous activation of HIF2α in pancreatic cells resulted in severe abnormalities in the postnatal pancreas including loss of acinar cells, acinar-ductal metaplasia, increased vascularization, and fibrosis.

To specifically activate HIF2α in the pancreas, we used the *Pdx1-Cre* transgenic line. *Pdx1* is expressed during early stages of the developing pancreatic epithelium and HIF2α is expressed in pancreatic progenitor cells^24^. Indeed, HIF2α inactivation causes pancreatic hypoplasia indicating an important role for HIF2α in pancreas development^24^. However, newborn *Pdx1-Cre;HIF2dPA* pancreata did not display apparent pancreatic abnormalities thus indicating the HIF2α overactivation do not overtly affect embryonic development of the pancreas. After birth, a gradual loss of acinar cells was observed in *Pdx1-Cre;HIF2dPA* pancreata. Indeed, *Pdx1-Cre;HIF2dPA* mice develop features reminiscent of chronic pancreatitis including acinar cell loss, acinar-ductal metaplasia, inflammatory cell infiltration, fibrosis and lipomatosis. Despite the extensive acinar cell loss, *Pdx1-Cre;HIF2dPA* mice did not display loss of body weight after birth. However, it should be noted that the loss of pancreatic enzyme synthesis needs to be almost complete to cause overt physiological defects^44,45^.

Concomitant with the acinar cell loss, a marked increase in duct-like tubular structures was observed in *Pdx1-Cre;HIF2dPA* pancreata. Acinar-ductal metaplasia is commonly observed in chemical models of pancreatic injury as well as in several mutant mice deficient in genes critical for exocrine cell homeostasis and it is considered an adaptive response to acinar cell injury^46,47^. Although increased acinar cell proliferation was prominent in *Pdx1-Cre;HIF2dPA* mice, this did not result in tissue regeneration. Indeed, two-month-old *Pdx1-Cre;HIF2dPA* mice displayed a massive loss of acinar cells indicating that sustained HIF2α was detrimental to acinar cell regeneration. Lineage tracing experiments have demonstrated transdifferentiation of acinar cells into ductal-like cells in several animal models of acinar cell injury (reviewed in^47,48^). In our study, lineage tracing could not be utilized to determine the acinar origin of ductal-like cells in *Pdx1-Cre;HIF2dPA* pancreata because the *Pdx1-Cre* transgene is expressed in common pancreatic progenitors. We did not detect intermediate cells co-expressing acinar and ductal markers in *Pdx1-Cre;HIF2dPA* pancreata that could suggest acinar-duct transdifferentiation but this is just circumstantial evidence. We did observe increased cell apoptosis in postnatal *Pdx1-Cre;HIF2dPA* pancreata. Thus, apoptosis could account for the severe loss of acinar cells although we cannot rule out an additional contribution from transdifferentiation of acinar cells into ductal-like cells. Although HIF2α usually promote cell survival and proliferation, a role of HIF2α in inducing cell apoptosis has also been reported. For example, HIF2α overexpression in hepatocellular carcinoma^49^ and glioblastoma cells^50^ increases tumor cell apoptosis. The proapoptotic effect of HIF2α overexpression on acinar cells could also be indirect as a response to acinar cell injury, as observed in pancreatitis. In agreement with this notion, we observed expression of the injury marker clusterin in *Pdx1-Cre;HIF2dPA* pancreata.

The duct-like tubular structures of *Pdx1-Cre;HIF2dPA* mice are reminiscent of the cysts and microcystic adenomas commonly found in VHL patients^10,11^, which are characterized by increased vascularization and prominent fibrous stroma. However, most pancreatic lesions in VHL patients are asymptomatic and no extensive acinar cell loss is usually found. Only rare cases of pancreatitis have been reported and these appear to be secondary to obstruction of the main pancreatic duct of cysts. A potential explanation for the difference in pancreatic phenotypes between VHL patients and *Pdx1-Cre;HIF2dPA* mice is that HIFα activation (by VHL inactivation) occurs locally in VHL patients (second hit hypothesis of hereditary cancer) while broad HIFα activation in the pancreas is achieved in *Pdx1-Cre;HIF2dPA* mice. In agreement with this notion, pancreas-specific *Vhlh* (the murine homolog of *VHL*) inactivation in mice results in the formation pancreatic cysts, loss of exocrine pancreas and fat replacement^21^. Interestingly, these pancreatic lesions were observed only in older *Vhlh* mutant mice, not in young mice^21^, as we observed in *Pdx1-Cre;HIF2dPA* mice. This apparent discrepancy might be related to the specificity and/or levels of HIF2α activation obtained in our mouse genetic model. Nonetheless, it is important to note that *Vhlh* mutant mice display severe perinatal lethality^15,21^ and thus, the analysis of pancreata in older *Vhlh* mutant mice was limited to few surviving mice^21^, which could have not achieved full HIF activation on exocrine pancreas. In this regard, it would be interesting to determine which specific HIF transcription factors are activated in the pancreatic lesions of VHL patients. *Vhlh* mutant mice exhibit impaired glucose homeostasis including severe hypoglycemia and defective insulin and glucagon secretion. Although we did not perform a complete characterization of glucose homeostasis, *Pdx1-Cre;HIF2dPA* mice showed normal fed glucose levels. This apparent discrepancy might be due to differences in the specific HIFα overactivated between *Pdx1-Cre;HIF2dPA* and *Vhlh* mutant mice. In support of this hypothesis, the abnormal glucose homeostasis of ß-cell-specific *Vhlh* mutant mice is HIF1α-dependent since deletion of HIF1α restores impaired glucose homeostasis^12,16^. Thus, our results suggest that HIF2α overactivation in the pancreas do not markedly alter glucose homeostasis.

The pancreatic defects of *Pdx1-Cre;HIF2dPA* mice are similar to those observed in mice with defects in primary cilia formation and it has been shown that pVHL contributes to cilia maintenance and stability in kidney cells^51–53^. Indeed, cilia are lost in kidney cysts from VHL patients^53^. To the best of our knowledge, it has not been reported whether pancreatic cysts in VHL patients lack primary cilia^54^. However, primary cilia were clearly present on the ductal cells of *Pdx1-Cre;HIF2dPA* pancreata indicating that HIF2α overactivation does not overtly compromise primary cilia integrity. Interestingly, it has been reported that only HIF1α (not HIF2α) activity is important for cilia formation^53^.

The loss of acini organization in *Pdx1-Cre;HIF2dPA* mice was associated with alteration of the MAPK and AKT/mTOR signaling pathways. These pathways play a pivotal role in acinar cell homeostasis in response to injury. Thus, MAPK signaling is required for the dedifferentiation of acinar cells into duct- like cells^39^. In concordance with this notion, upregulation of pERK signal was observed in acinar cells located in disorganized acini of *Pdx1-Cre;HIF2dPA* mice. Interestingly, we also observed prominent MAPK activity in the stromal cells of *Pdx1-Cre;HIF2dPA* pancreata, a phenomenon also reported in fibrosis associated to pancreas cancer formation^55^. Disorganization of acini structure was also accompanied by decreased levels of phosphorylated RPS6, a downstream target of the AKT/mTOR signaling pathway that has been implicated in acinar-ductal metaplasia^40–42^. Crosstalk between these signaling pathways and HIFs have been reported^23,56^, thus providing a potential causal link between HIF2α overactivation and impairment of acinar cell homeostasis.

Depletion of *Ptf1a*, a master regulator of acinar differentiation and function in adult mice also results in decreased levels of phosphorylated RPS6 in acinar cells concomitant with loss of acinar cell identity^43^. Indeed, the postnatal pancreatic exocrine degeneration observed in *Pdx1-Cre;HIF2dPA* mice is similar to defects observed in mutant mice deficient in transcription factors critical for acinar cell formation and identity such as MIST1, NR5A2, GATA6 and PTF1A^28,29,43,57^. *Pdx1-Cre;HIF2dPA* pancreata displayed features compatible with loss of acinar cell identity including decreased expression of amylase, disruption of acini organization, mislocalization of acinar cell apical markers and increased expression of ductal markers. However, these phenotypes could be secondary effects of acinar cell injury, as reported in animal models of pancreatitis. Temporal, synchronous activation of HIF2α in adult acinar cells would be necessary to elucidate the direct role of HIF2α in acinar cell homeostasis.

A potential role for HIF2α in pancreatic cancer formation has been recently described. HIF2α is highly expressed in preneoplastic lesions but is downregulated during malignant progression of pancreatic cancer^20^. HIF2α inactivation impairs the progression of cancerous lesions in the Kras-mediated mouse model of pancreatic cancer^20^. We did not observe tumor formation in *Pdx1-Cre;HIF2dPA* mice but our analysis was limited to relatively young mice (two-month-old mice). Future studies in genetic models of pancreatic cancer will help to determine the effect of HIF2α activation in pancreatic cancer.

## Methods

### Mice

Mice were housed in a pathogen-free facility at the Institute of Biomedicine of Seville (IBiS). All procedures involving experimental animals were performed in accordance with European and local animal welfare laws, guidelines and policies. All animal studies were approved by the IBiS-Virgen del Rocio Ethics Committee. *Pdx1-Cre* and *Hif2dPA* (*Rosa26Sor*^Tm4(Hif2A*)Kael^) mice carrying a floxed allele of human *HIF2a* have been previously described^25,26^. *Hif2dPA* mice were purchased from Jackson Labs.

### Histological and immunohistochemical analyses

Histological (including alcian blue, periodic acid-Schiff and Gomori’s one-step trichrome staining) as well as immunohistochemical analyses were performed as previously described^58,59^. Fluorescent and bright-field images were captured using a BX-61 microscope (Olympus) and LTC SP2 confocal microscope (Leica). All photomicrographs shown are representative of at least 3 independent samples of the indicated genotype. Primary antibodies were used at the indicated dilution: Rabbit anti-cleaved caspase-3 (1:50, 9661), rabbit anti-phosphorylated-ERK (1:300, 4370) and rabbit anti-phosphorylated-S6 ribosomal protein (1:75, 4858) from Cell Signaling Technology; rabbit anti-carboxypeptidase A (1:200, 1810-0006) from AbD Serotec; mouse anti-E-cadherin (1:200, 610181), rat anti-panendothelial cell antigen (MECA32) (1:300, 553849) and mouse anti-β-catenin (1:300, 610153) from BD Pharmigen; rat anti-F4/80 (1:50, T-2006; BMA Biomedicals); rat anti-cytokeratin 19 (1:100, TROMA-III; Developmental Studies Hybridoma Bank); rabbit anti-ZO-1 (1:100, 40-2300; Invitrogen); rabbit anti-Sox-9 (1:3000, AB5535; Merck Millipore); rabbit anti-CD11b (1:200, NB110-89474) and rabbit anti-HIF2α diluted 1:100 (NB100-122) from Novus; mouse anti-amylase (1:200; Sc-46657) goat anti-α-clusterin (1:200, sc-6419) and rabbit anti-PKCε (1:100, sc-214) from Santa Cruz Biotechnology; mouse anti-acetylated tubulin (1:10000, T-6793), mouse anti-α-smooth muscle actin (1:1000, A-5228), rabbit anti-laminin (1:500, L-9393) and rabbit anti-vimentin (1:1000, AB92547) from Sigma-Aldrich and armenian hamster anti-Mucin-1 (1:200, HM-1630; Thermo Scientific). The biotin conjugate of the lectin *Dolichos biflorus* agglutinin was obtained from Vector Laboratories and detected with streptavidin-conjugated FITC (Jackson ImmunoResearch).

To quantify cell proliferation, following double immunofluorescence for the proliferation marker Ki-67 (RM-9061; Thermo Scientific) and amylase or KRT19, positive cells were then counted from 10 non-overlapping fields at 200x magnification from 3 control and 3 *Pdx1-Cre;Hif2dPA* mice. Cell apoptosis was detected using *In Situ* Cell Death Detection Kit (Roche) according to the manufacturer’s protocol in 10 non-overlapping fields at 200x magnification from 4 control and 5 *Pdx1-Cre;Hif2dPA* mice. Pancreatic sections treated with DNAse I (Sigma-Aldrich) were used as positive control. Acinar cell apoptosis was quantified by averaging the number of TUNEL-positive cells per total number of amylase-positive cells. Morphometric analysis of vimentin, CD11b and F4/80 were performed by using ImageJ software. For CD11b and F4/80, the number of positive cells per field was calculated. The extent of vimentin accumulation was measured as the percentage of vimentin-positive area per total pancreatic area. For each group, 10 randomly selected low-power fields from pancreatic sections at least 60 µm apart in three mice were analyzed.

### Western Blot analysis

Tissue samples were lysed and homogenized in lysis solution (50 mM Tris-HCl [pH 7.4], 100 mM NaCl, 5 mM MgCl_2_, 0,1% SDS, 1% Triton X-100) and Complete Ultra protease inhibitors (Roche). Protein concentration was determined using the Pierce BCA Protein Assay Kit (Thermo Fisher Scientific). After SDS-PAGE separation, samples were transferred to PVDF membranes (BioRad), processed for immunoblotting against anti-HA (1:500, 1167423001; Roche) and anti ß-actin (1:1000, ab8226; Abcam) antibodies, developed using HRP-conjugated secondary antibodies (Jackson) and Western ECL Substrate (Bio-Rad). ImageQuant LAS 4000 Mini Gold (GE Healthcare) was used for imaging the blots.

### RNA isolation and Quantitative Real Time PCR

RNA isolation was performed using RNeasy Kit (Qiagen) with deoxyribonuclease treatment, following manufacturer’s instruction. Total RNA purity and concentration was assessed using Nanodrop 2000 spectrophotometer (Thermo Scientific, Wilmington, NC, USA), and subsequently reverse-transcribed using random hexamer primers and the cDNA Omniscript reverse transcriptase (Qiagen). Quantitative PCR was performed using with SYBR Green PCR Master Mix (Applied Biosystems) using a 7900HT real-time PCR system (Applied Biosystems). Relative quantification of RNA levels was calculated using the ΔΔCt method. Cyclophilin A (peptidylprolyl isomerase A-Mouse Genome Informatics) was used as housekeeping gene^59^. The results were expressed as fold relative to levels in control pancreata (value of 1). Primer sequences for cyclophylin A and VEGF were as follows: CycloA-forward 5′-TCACAGAATTATTCCAGGATTCATG-3′; CycloA-reverse 5′-TGCCGCCAGTGCCATT-3′; VEGF-forward 5′-CCAGCACATAGGAGAGATGAGCTT-3′; VEGF-reverse 5′-TCTGTCTTTCTTTGGTCTGCATTC-3′.

### Statistical analysis

Significance was determined using two-tailed Student’s t-test, and one-way ANOVA (post-hoc Tukey HSD test). *P* < 0.05 was considered significant. Data are presented as mean ± SD.

## Electronic supplementary material

Supplementary information

## Data Availability

All data generated or analysed during this study are included in this published article.
