# Supplementary material for: Stabilization of HIF-2α impacts pancreas growth
Source: Sci Rep. 2018 Sep 12;8:13713. doi: 10.1038/s41598-018-32054-5 (PMC6135861; doi:10.1038/s41598-018-32054-5)
Supplement: Supplementary file 1 — Supplementary information [file 41598_2018_32054_MOESM1_ESM.docx]

**Stabilization of HIF-2α impacts pancreas growth.**

Alvaro Flores-Martínez^1#^, Alejandro García-Núñez^1#^, Anabel Rojas^2,3^ and David A. Cano^1*^

^1^Unidad de Gestión de Endocrinología y Nutrición. Instituto de Biomedicina de Sevilla (IBiS), Hospital Universitario Virgen del Rocío/CSIC/Universidad de Sevilla, Spain.

^2^Centro Andaluz de Biología Molecular y Medicina Regenerativa

CABIMER- Universidad Pablo de Olavide- Universidad de Sevilla-

Consejo Superior de Investigaciones Científicas (CSIC), Sevilla, Spain

^3^Centro de Investigación Biomédica en Red de Diabetes y Enfermedades

Metabólicas Asociadas (CIBERDEM), Madrid, Spain

^#^ These authors contributed equally to this work.

**Author for correspondence:** David A. Cano

^1^Unidad de Gestión de Endocrinología y Nutrición. Instituto de Biomedicina de Sevilla (IBiS), Consejo Superior de Investigaciones Científicas, Universidad de Sevilla. Avda. Manuel Siurot, s/n. Hospital Universitario Virgen del Rocío, 41013 Sevilla, Spain.

e-mail: dcano-ibis@us.es


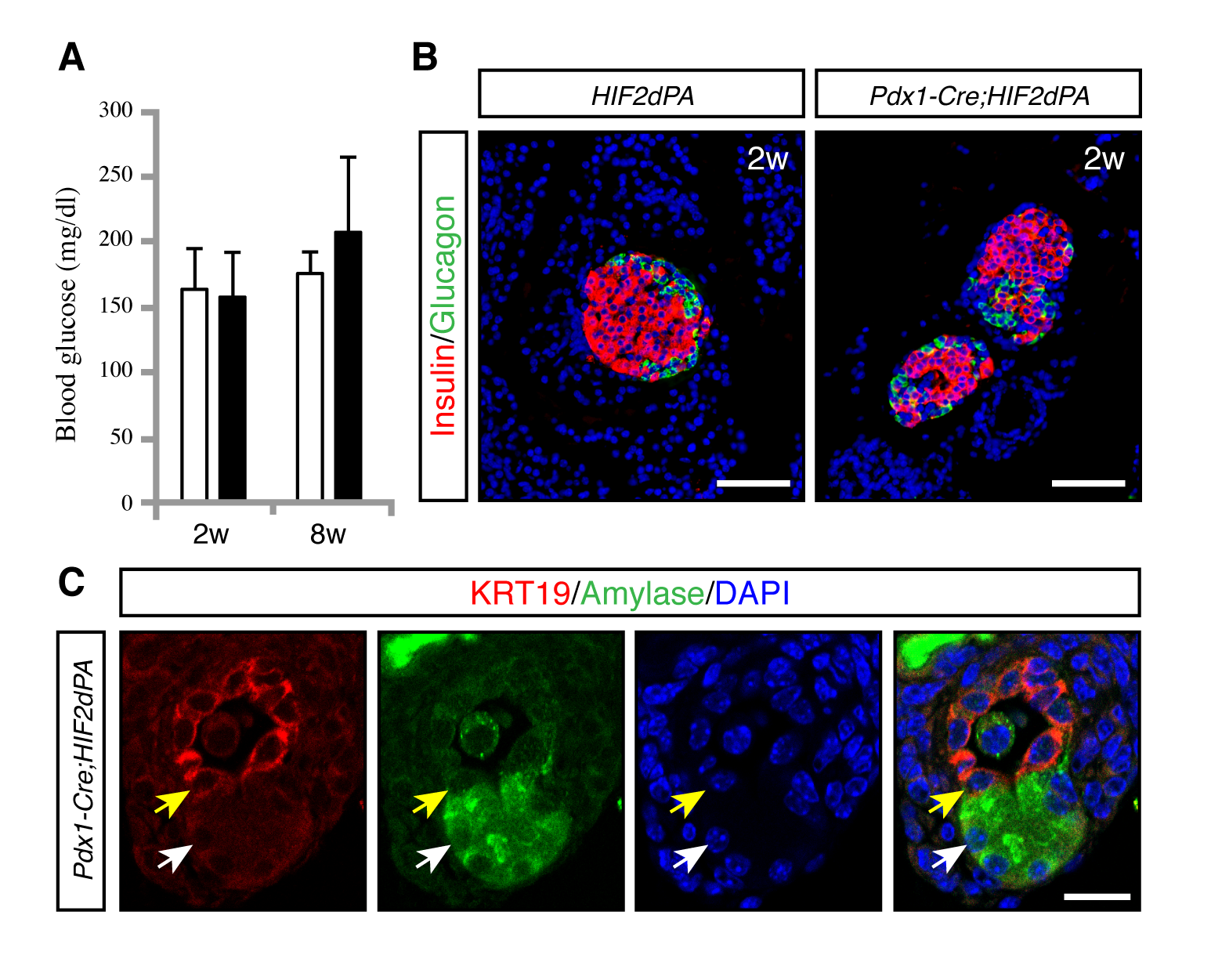


**Supplementary Figure 1. Islet architecture and acinar-ductal metaplasia in HIF2α-overexpressing mice.** (A) Fed glucose levels in *Pdx1-Cre;HIF2dPA* and control mice at 2 and 8 weeks of age. (B) Normal islet architecture in two-week-old *Pdx1-Cre;HIF2dPA* mice compared to control mice. (C) Confocal microscope images of an area of acinar-ductal metaplasia in *Pdx1-Cre;HIF2dPA* pancreas showing the absence of amylase and KRT19 colocalization. Yellow arrows indicate KRT19-positive cells and white arrows indicate amylase-positive cells. Nuclei are stained with DAPI (blue). Scale bars= 50 µm for B; 20 µm for C.


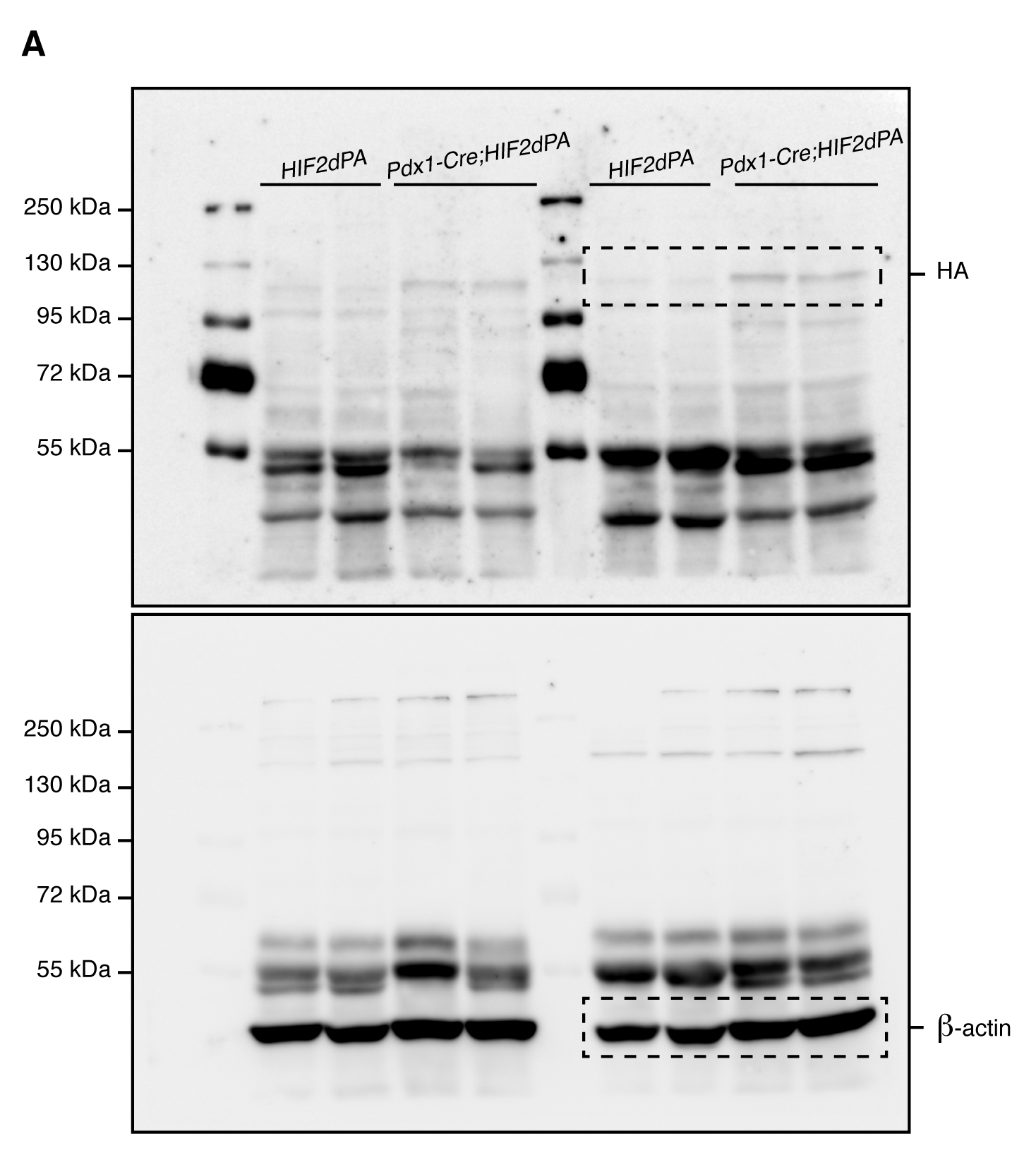


**Supplementary Figure 2. Full-length blots of western blot analysis.** (A) Full-length blots of western blot analysis shown in Figure 1B. Efficient HA accumulation in *Pdx1-Cre;HIF2dPA* pancreata. β-actin protein was used for loading control. Four independent two-week-old control and mutant mice are shown. First and sixth lanes from the left are PageRuler Plus prestained protein ladder (Thermo Scientific). Black dotted lines indicate images shown in Figure 1B.
